# Identification of a large intronic transposal insertion in *SLC17A5* causing sialic acid storage disease

**DOI:** 10.1186/s13023-017-0584-6

**Published:** 2017-02-10

**Authors:** Maja Tarailo-Graovac, Britt I. Drögemöller, Wyeth W. Wasserman, Colin J. D. Ross, Ans M. W. van den Ouweland, Niklas Darin, Gittan Kollberg, Clara D. M. van Karnebeek, Maria Blomqvist

**Affiliations:** 1 BC Children’s Hospital Research Institute, University of British Columbia, Vancouver, BC Canada; 2 Department of Medical Genetics, University of British Columbia, Vancouver, Canada; 3 Centre for Molecular Medicine and Therapeutics, Vancouver, Canada; 4 Pharmaceutical Sciences, University of British Columbia, Vancouver, BC Canada; 5 Department of Clinical Genetics, Erasmus Medical Center, Rotterdam, The Netherlands; 6 Department of Pediatrics, Sahlgrenska Academy, Gothenburg University, Gothenburg, Sweden; 7 Department of Clinical Chemistry and Transfusion Medicine, Institute of Biomedicine, Sahlgrenska Academy, University of Gothenburg, Gothenburg, Sweden; 8 Department of Pediatrics, University of British Columbia, Vancouver, Canada; 9 Department of Pediatrics, Academic Medical Centre, Amsterdam, The Netherlands

**Keywords:** Sialic acid storage disease, Salla disease, *SLC17A5*, Whole exome sequencing, Transposon insertion

## Abstract

**Background:**

Sialic acid storage diseases are neurodegenerative disorders characterized by accumulation of sialic acid in the lysosome. These disorders are caused by mutations in *SLC17A5*, the gene encoding sialin, a sialic acid transporter located in the lysosomal membrane. The most common form of sialic acid storage disease is the slowly progressive Salla disease, presenting with hypotonia, ataxia, epilepsy, nystagmus and findings of cerebral and cerebellar atrophy. Hypomyelination and corpus callosum hypoplasia are typical as well. We report a 16 year-old boy with an atypically mild clinical phenotype of sialic acid storage disease characterized by psychomotor retardation and a mixture of spasticity and rigidity but no ataxia, and only weak features of hypomyelination and thinning of corpus callosum on MRI of the brain.

**Results:**

The thiobarbituric acid method showed elevated levels of free sialic acid in urine and fibroblasts, indicating sialic acid storage disease. Initial Sanger sequencing of *SLC17A5* coding regions did not show any pathogenic variants, although exon 9 could not be sequenced. Whole exome sequencing followed by RNA and genomic DNA analysis identified a homozygous 6040 bp insertion in intron 9 of *SLC17A5* corresponding to a long interspersed element-1 retrotransposon (KF425758.1). This insertion adds two splice sites, both resulting in a frameshift which in turn creates a premature stop codon 4 bp into intron 9.

**Conclusions:**

This study describes a novel pathogenic variant in *SLC17A5*, namely an intronic transposal insertion, in a patient with mild biochemical and clinical phenotypes. The presence of a small fraction of normal transcript may explain the mild phenotype. This case illustrates the importance of including lysosomal sialic acid storage disease in the differential diagnosis of developmental delay with postnatal onset and hypomyelination, as well as intronic regions in the genetic investigation of inborn errors of metabolism.

**Electronic supplementary material:**

The online version of this article (doi:10.1186/s13023-017-0584-6) contains supplementary material, which is available to authorized users.

## Background

The primary catabolic pathway of sialo-glycoconjugates is lysosomal degradation. In the lysosome, sialic acid residues are sequentially removed from the carbohydrate chain by sialidases (neuraminidases), and then transported out of the lysosome by the transporter protein sialin [1, 2]. Sialin is a member of the SLC17 solute carrier family, a group of structurally related membrane proteins that is a part of the major facilitator superfamily of transporters [3, 4]. The SLC17 family proteins have diverse crucial biological functions and several members are associated with inherited neurological or metabolic diseases [2].

Sialic acid storage diseases (SASDs) are autosomal recessive neurodegenerative disorders that are characterized by an excessive storage of sialic acid in the lysosomes caused by defective transport by the sialin protein. Two main disorders represent this group i.e. infantile sialic acid storage disease (ISSD, OMIM #269920) and the slowly progressive adult form that is prevalent in Finland called Salla disease (OMIM #604369) [5]. ISSD is a more severe form resulting in an early-lethal multisystemic disease. Common symptoms are intellectual developmental disorder, muscular hypotonia, failure to thrive as well as coarse features, seizures, bone malformations, hepatosplenomegaly, and cardiomegaly in some patients. Furthermore, this condition is associated with non-immune hydrops fetalis. Clinical symptoms of Salla disease include nystagmus, muscular hypotonia, ataxia, early psychomotor retardation and speech impairment. Epilepsy is a common feature and cerebral and cerebellar atrophy, hypomyelination and corpus callosum hypoplasia are typical findings on these patients. The clinical progression is slow, and patients can live throughout adulthood.

In 1999 Verheijen *et al* identified *SCL17A5* as the gene coding the sialin protein and found mutations in this gene in SASD patients [6]. Since then, a wide spectrum of disease-causing variants have been reported in *SLC17A5*, including missense and nonsense mutations, splice site mutations and deletions. Of particular relevance to individuals from Finland and Sweden is the relatively high frequency of the c.115C > T transition (NM_012434.4; rs80338794). This missense mutation changes a highly conserved arginine to a cysteine (p.Arg39Cys) and, in homozygous form, causes the classical slowly progressing Salla phenotype [7, 8]. The estimated carrier frequency for this variant in Finland is reported to be as high as 1/200 [7]. Other *SLC17A5* mutations that have more damaging effects on the sialin protein function cause ISSD. Individuals diagnosed with intermediate Salla disease usually have one allele of p.Arg39Cys in compound heterozygosity with a more severe mutation [7].

As a result of the excessive storage of sialic acid, SASD patients excrete large amounts of free sialic acid in their urine. Thus this biomarker is used for biochemical diagnosis of these patients. Classical Salla patients excrete about 10 times more free sialic acid in urine compared to healthy controls and in ISSD patients this elevation is even higher (about 100 times the normal levels). Furthermore, the storage of sialic acid can also be detected in tissue samples and cultured fibroblasts.

This report describes a new intronic transposal insertion in *SLC17A5* in a patient showing elevated free sialic acid in urine and fibroblasts, giving rise to a milder phenotype of SASD than ISSD and Salla disease. The results further highlight the importance of expanding molecular analyses to non-coding regions when biochemical signs point towards a certain diagnosis.

## Methods

### Clinical report

**Fig. 1:**
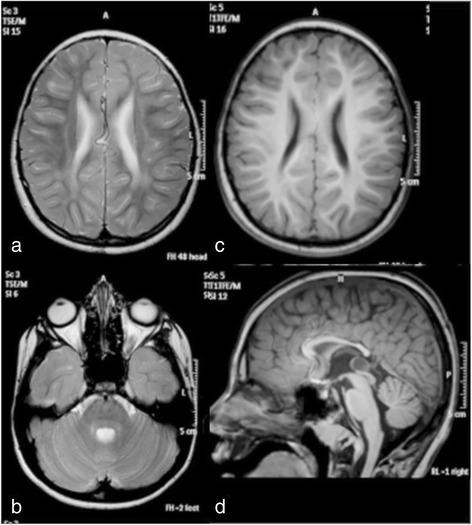
Axial T2 sequences showed slightly increased T2 signal in supratentorial central white matter (**a**) while the cerebellar white matter looked normal (**b**). Axial T1-weighted imaging showed normally signaling supratentorial white matter (**c**). Sagittal T2-weighted imaging revealed a somewhat thin corpus callosum and a small cyst (1.2 cm) of the corpus pineale (**d**)

This boy is the first child to healthy unrelated parents of Kurdish origin. Apart from transiently decreased fetal movements reported by the mother (during 7th months of pregnancy), the pregnancy was uneventful. The boy was born after 37 weeks of gestation with a birth weight of 2630 g, length of 49 cm and head circumference of 32 cm. The Apgar score was 10-10-10 and the perinatal period was uncomplicated. The boy was first admitted at 9 months of age because of bilateral esotropia. He was then also found to have hyperopia (+7-8). Regular ophthalmological investigation revealed no other changes. He was again admitted at 13 months of age because of delayed psychomotor development. He could sit, grasp with his whole hands and move between them and babbled with two syllables, corresponding to a developmental age of around 6–7 months. Muscle tone and tendon reflexes were normal. He learned to say a few words and to walk unsupported around 3 years of age. His communicative skills peaked at 5 years of age when he could combine 2–3 words, while his best motor function was at 9 years of age when he could walk unsupported both uphill and downhill. He has since then slowly lost developmental skills and has become increasingly stiff. A trial of L-Dopa treatment had no effect. A permanent gastrostomy was placed at 11 years of age because of swallowing difficulties although he still managed to eat small pieces of food by himself. At the last assessment at 16 years of age, he was a very happy and easy-going young man with the ability to communicate with gestures, sounds, pointing and about 30 signs and a few words. He was able to walk around 100 m with support and had a comparably good fine motor function with bilateral pincer grasp. On examination he had generally increased muscle tone considered to be a mixture of spasticity and rigidity with associated contractures in large joints and a right-sided scoliosis. The muscle tendon reflexes were generally increased with left-sided ankle clonus and a right-sided Babinski’s sign. There were no involuntary movements or signs of ataxia. He had no seizures and EEG was normal. Head circumference and height has been normal and there were no signs of additional organ involvement. Routine laboratory investigations including metabolic screening analyses have been normal. A 3 T MRI of the brain was performed at 8 years of age showing mild features of hypomyelination and thinning of corpus callosum (Fig. 1). In view of the unusual phenotype, further investigation of the *SLC17A5* gene was driven by the elevation of free sialic acid in urine and fibroblasts.

### Materials

Skin fibroblasts were obtained and cultured according to routine procedures in Eagle’s minimal essential medium supplemented with 10% foetal calf serum and 1% PEST. Confluent cells were harvested by trypsination and stored in -20 °C until analysis.

Urine samples were collected as morning aliquots and stored at -20 °C until analysis.

### Analysis of sialic acid

Fibroblasts were suspended in water and homogenized using a glass/teflon homogenizer. After addition of sodium chloride to a final concentration of 0.15 M, the cells were centrifuged at 20 000xg for 30 min at +4 °C and the supernatant was collected for determination of free sialic acid (see below). Cell protein concentration was determined by the BCA method (Pierce laboratories). Analysis of free sialic acid in urine was performed by thin-layer chromatography. The plates were developed in 1-butanol/acetic acid/water (2:1:1 by volume) and visualized with resorcinol reagent. Total (free and bound) sialic acid in urine was determined with the resorcinol method [9] after hydrolysis by 0.05 M sulphuric acid and purification with ion-exchange chromatography. Free sialic acid in urine and fibroblasts were analyzed by the thiobarbituric acid method according to Aminoff [10] after purification by ion-exchange chromatography.

### Sanger sequencing of SLC17A5

Mutation analysis of all coding exon (including 30 nucleotides before and after the exon) of *SLC17A5* was performed by Sanger Sequencing (primers available on request) using an ABI 3730XL automated sequencer (Applied Biosystems, Foster City, CA, USA). Data were analysed using SeqPilot software (version 4.2.1 build 506; JSI medical systems GmbH, Ettenheim, Germany).

### Whole exome sequencing

Given the mild elevation of sialic acid and the results from the initial Sanger sequencing of the coding exons of *SLC17A5*, a novel inborn error of metabolism was suspected and thus whole exome sequencing (WES) was performed through the TIDEX gene discovery project (UBC IRB approval H12-00067). WES was performed for the index and his unaffected parents using the Agilent SureSelect kit and Illumina HiSeq 2000 (Perkin-Elmer, USA). The data was analyzed using our semi-automated bioinformatics pipeline [11]. Briefly, the sequencing reads were aligned to the human reference genome version hg19 and rare variants were identified and assessed for their potential to disrupt protein function, and subsequently screened under a series of genetic models: homozygous, hemizygous, compound heterozygous and *de novo*.

### SLC17A5 RNA/cDNA analysis

Total RNA was extracted from the cultured skin fibroblasts using the AllPrep DNA/RNA Mini kit (Qiagen). RT-PCR was performed with the One-step RT-PCR kit (Qiagen) with forward primer 5’-TATTCCTGGTAGCTGCTGGC-3’ and reverse primer 5’- TCTGGCAACTAGTGATATTTCATGA-3’ predicted to amplify a product of 517 bp in length of NM_012434.4; SLC17A5 cDNA (c.1130 – c.1646).

PCR products were separated on agarose gels stained with GelStar® and visualized on a Dark Reader Blue light transilluminator. Sequencing analysis was performed using an ABI PRISM® 3100 Genetic Analyzer and the BigDye Terminator v.1.1 Cycle Sequencing Kit (Applied Biosystems).

### PCR and Sanger sequencing of the genomic DNA

Primers were designed using the *SLC17A5* DNA reference sequence (Ensembl Gene ID ENST00000355773). An initial long-range PCR was performed using primers spanning the suspected location of the insertion (forward primer: 5’ - CTT CTG GAT TTA GCA TCA ACC A - 3’ and reverse primer: 5’ - AGT ATT CCT GGT AGC TGC TG – 3’). The resultant PCR products were used as templates for the following nested PCRs:

i) A long range PCR (forward primer: 5’ - CTT CTG GAT TTA GCA TCA ACC A - 3’ and reverse primer: 5’ - CAA CTT CCT GCT TTA ATT ATT GTG – 3’) to determine the location and sequence of the identified insertion using Sanger sequencing
ii) A PCR-based assay to confirm the presence/absence of the insertion using two primers outside of the insertion (forward primer: 5’ - CTT CTG GAT TTA GCA TCA ACC A - 3’ and reverse primer: 5’ - CAA CTT CCT GCT TTA ATT ATT GTG – 3’) and a third primer located inside of the insertion (forward primer: 5’ - AAT ATT CGG GTG GGA GTG AC – 3’).

Sanger sequencing was performed using BigDye® Terminator v3.1 Cycle Sequencing chemistry (Life Technologies) and subsequent capillary electrophoresis was performed by the CMMT/CFRI DNA Sequencing Core Facility using a Prism 3130xl 16-capillary automated genetic analyzer (Applied Biosystems). *In silico* analyses to determine the class of transposon and the effect on splicing were performed using (i) RepeatMasker [12] and (ii) NetGene2 [13] and Alternative Splice Site Predictor (ASSP) [14].

## Results

### Sialic acid

Sialic acid (total and free) was analyzed twice when the patient was 11 years old (Table 1). While total sialic acid in urine was borderline normal, thin-layer chromatography showed an abnormal pattern with large amounts of free sialic acid (data not shown). Subsequent quantitative analysis revealed elevated amounts of free sialic acid in urine and fibroblasts (Table 1).

**Table 1:** Sialic acid in urine and fibroblasts

Analyte	Sampling 1	Sampling 2	Normal range
Sialic acid (total)—urine nmol/mol creatinine	68	69	31–69
Sialic acid (free) urine nmol/mol creatinine	60	57	7–21
Sialic acid (free) fibroblasts nmol/mg protein	16	-	<1,3

### Sanger sequencing of the coding regions of *SLC17A5*

In the first attempt to find pathogenic variants in the *SLC17A5* gene, Sanger sequencing of the coding regions was performed not showing any pathogenic variants, although exon 9 could not be sequenced (data not shown).

### Whole exome sequencing

Using our semi-automated bioinformatics approach [11], 20 candidate genes affected by rare variants predicted to affect protein function and segregating according to Mendelian inheritance models were identified. Based on inheritance patterns, these could be grouped into: homozygous (*SPTY2D1*, *LRP2* and *ERCC5*), hemizygous (*FLNA*, *ZNF275*, *GRIPAP1*, *AMER1*, *PLXNB3*, *TAS2R43*, *ARSH*, *MAGEA11* and *NLGN4X*), compound heterozygous (*PLXND1*, *NHSL1*, *COBL*, *PIEZO1*, *NUP153* and *MYH7B*) and *de novo* (*TCTE1* and *PUSL1*). However, none of these candidate genes could reasonably be assumed to cause the biochemical and clinical phenotype of the index. Interestingly, in addition to these, the WES analysis revealed a presence of clustered mismatches indicative of a homozygous insertion of an unknown size and origin in intron 9 of *SLC17A5*, with one of the breakpoints located 24 bp from the intron/exon boundary. Given the previously described spectrum of Salla disease-causing variants in *SLC17A5* and the fact that exon 9 could not be amplified by Sanger sequencing, a putative insertion was considered the best candidate and was further analyzed.

### SLC17A5 RNA/cDNA analysis

**Fig. 2:**
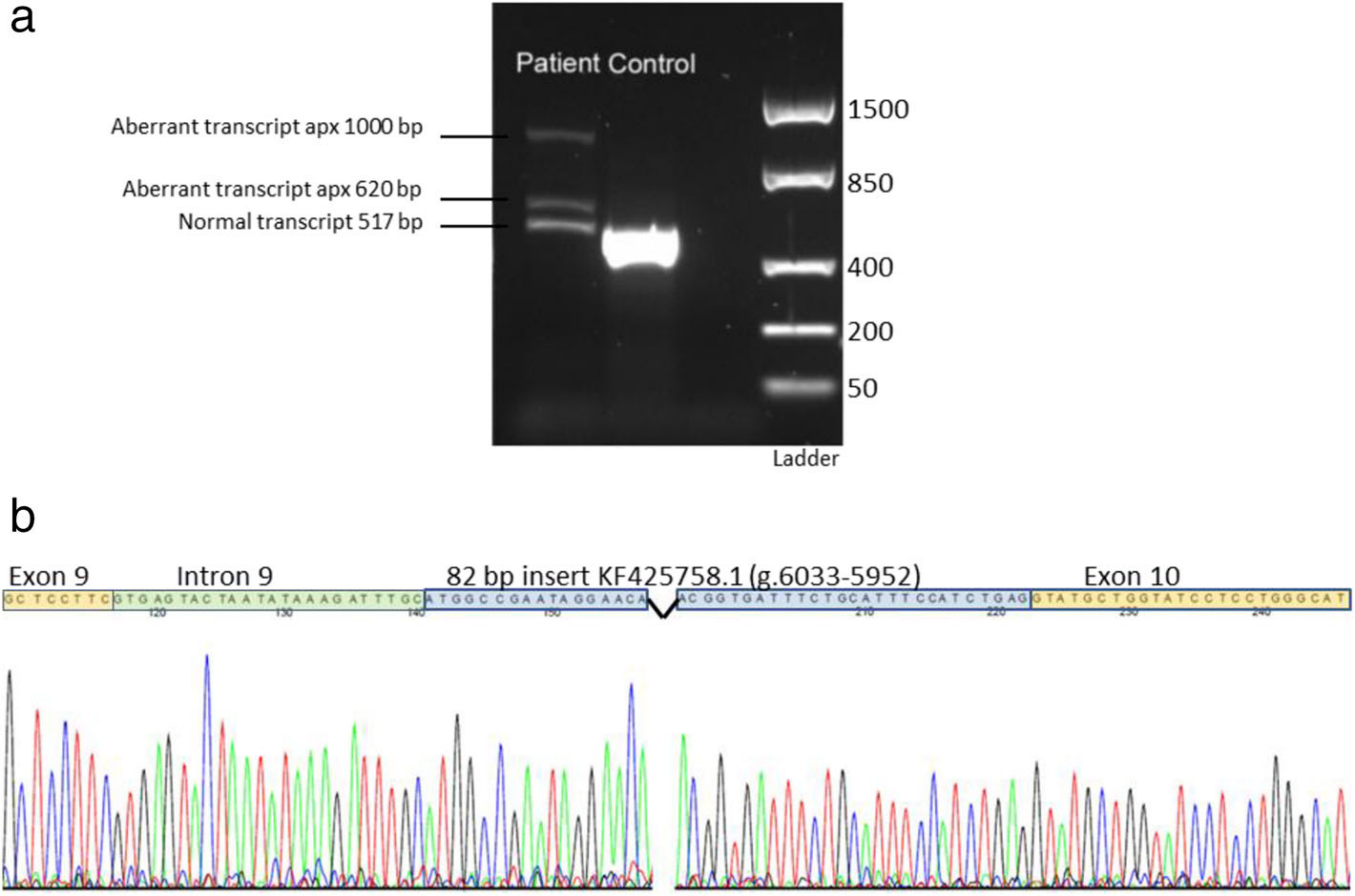
Genetic analyses. RT-PCR followed by gel electrophoresis of products spanning cDNA position c.1130 – c.1646 in the *SLC17A5* gene showing two extra fragments of abnormal size in addition to the expected 517 bp fragment in the patient. The abnormal transcripts were absent in the control sample (**a**). RNA was extracted from cultured skin fibroblasts. Direct sequencing of the 620 bp fragment revealed an apparently homozygous insertion of 106 bp in length, where the first 24 bp corresponded to an intronic sequence immediately adjacent to the exon 9 splice site, followed by an 82 bp sequence corresponding to position 6033-5952 of the transposable element KF425758.1 (**b**)

RT–PCR of *SLC17A5* cDNA from cultured skin fibroblasts, spanning cDNA c.1130 – c.1646, followed by gel electrophoresis, revealed three products instead of the expected single transcript of 517 bp in the patient but not in the control subject (Fig. 2a). One of the additional fragments was approximately 620 bp and the other about 1000 bp. All three bands were cut out and sequenced by Sanger sequencing. Sequencing analysis of the 620 bp fragment revealed an insertion 106 bp in length, where the first 24 bp corresponded to an intronic sequence immediately adjacent to the exon 9 splice site, followed by an 82 bp sequence corresponding to position 6033-5952 of a previously described transposable element KF425758.1 [15] (Fig. 2b). The sequencing data from the analysis of the > 1000 bp product was not readable due to too much noise and background. The 517 bp product corresponded to the reference sequence NM_012434.4 for both patient and control (not shown).

### PCR and Sanger sequencing of the genomic DNA

**Fig. 3:**
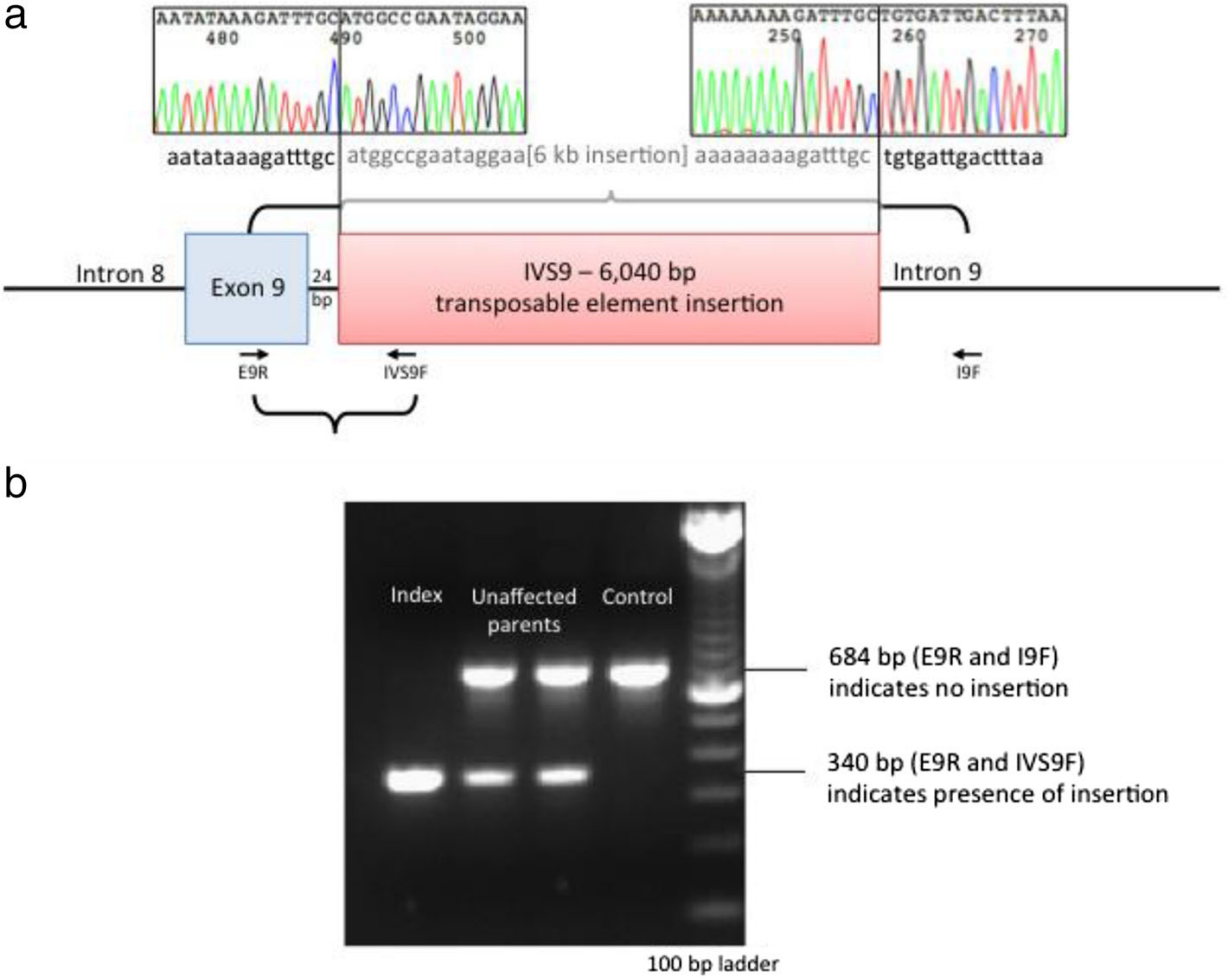
Sequencing of this ~6 kb fragment confirmed the presence of a 6040 bp LINE-1 retrotransposon, which was inserted in intron 9 (**a**). PCR analyses confirmed that the unaffected parents were heterozygous for this insertion, while the index was homozygous (**b**)

Subsequent Sanger sequencing of the region identified by the exome sequencing and RNA/cDNA analyses confirmed the presence of a 6040 bp insertion, which was located in intron 9 of *SLC17A5* (24 bp downstream of exon 9) (Fig. 3a). RepeatMasker analyses [16] revealed that this insertion was a long interspersed element-1 (LINE-1, L1) retrotransposon [17]. As expected based on the exome sequencing data and the RT-PCR analysis, the index was homozygous for this insertion, while the unaffected parents were heterozygous (Fig. 3b).

**Fig. 4:**
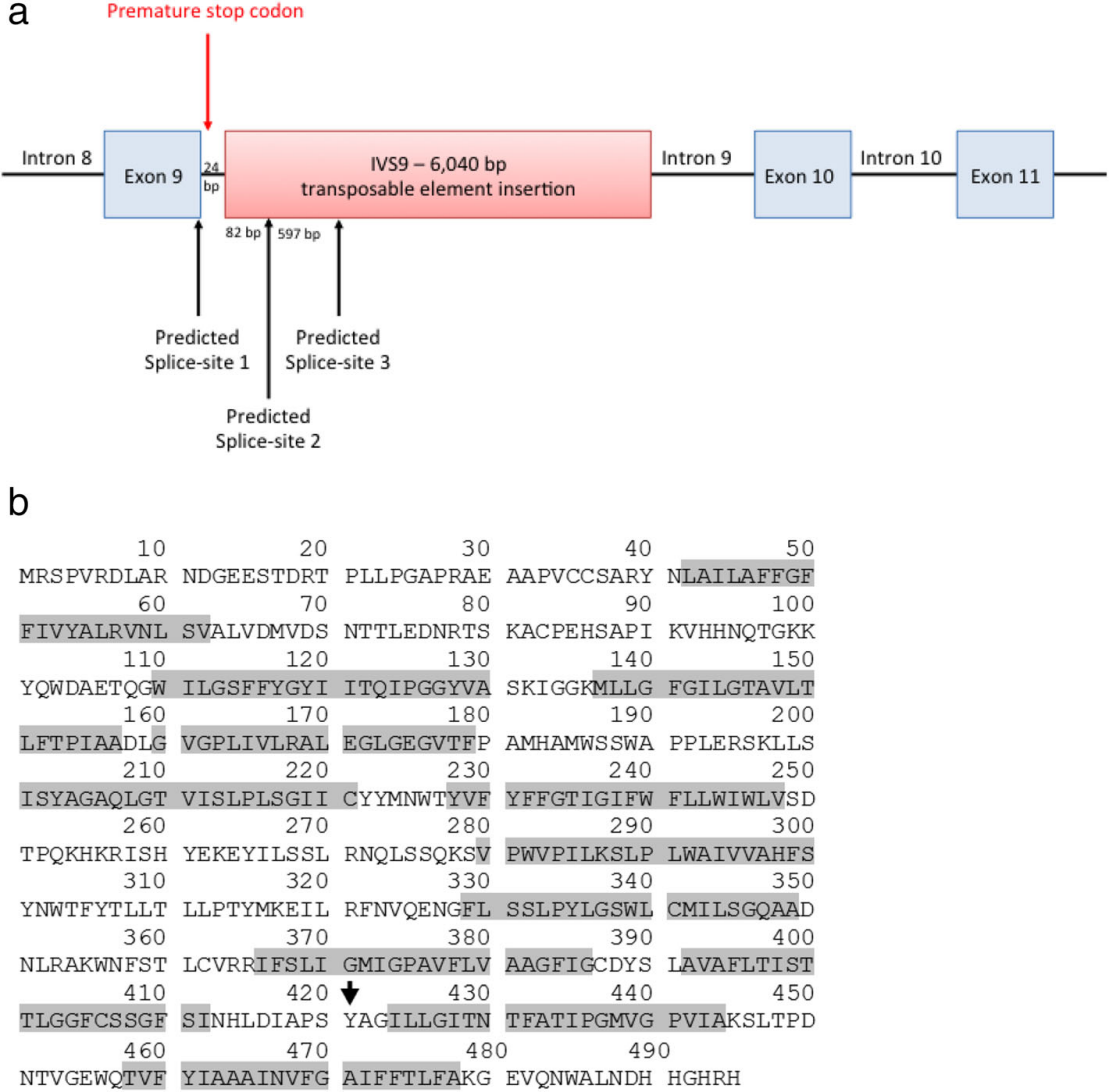
Splice site analyses using NetGene2 and ASSP, revealed that in the presence of the insertion, in addition to the correct splice-site occurring at the exon- intron boundary, a further two splice sites are present in the insertion (**a**). These alternate splicing events result in a frameshift mutation, which results in a premature stop codon at amino acid 421, indicated by the arrow (**b**). Grey shading indicated the 12 transmembrane regions of the sialin protein

In addition to the predicted splice site located at the exon-intron boundary, splice site analyses with NetGene2 revealed an additional predicted splice-site 82 bp into the insertion, while ASSP analyses identified a third predicted splice-site 679 bp into the insertion (Fig. 4a). The transcripts created by the three predicted splice sites would amplify a 517 bp fragment (wildtype), a 623 bp fragment and a 1220 bp fragment, in agreement with the cDNA analyses. The products from these two alternate splice-sites result in a frameshift, which causes a premature stop codon 4 bp into intron 9. In case of translation of the aberrant transcripts, the premature stop will be at amino acid 421 (Fig. 4b). The sequences for the resulting cDNA products are provided in the Additional file 1.

Investigation of structural variation in the 1000 Genome Project/Ensembl databases did not reveal any previous reports of this variant in healthy individuals.

## Discussion

The lysosomal free sialic acid storage disorders present with a broad clinical spectrum. Salla disease represents the mildest phenotype and occurs most frequently in Finland, and other Nordic countries such as Sweden and Denmark [7, 8, 18, 19]. The infantile form of sialic acid storage disease shows a more severe clinical phenotype and has no geographic predominance [7, 20, 21]. There also exists SASD forms that are intermediate in severity between Salla and ISSD [22–26]. Whereas Salla patients usually present with hypotonia, ataxia and nystagmus the first year of life, our patient showed delayed psychomotor development together with hyperopia at age 3 years. Ataxia usually remains a prominent feature as Salla disease progresses, however in the present case ataxia has not incurred. Instead, our patient shows increased muscle tone, most likely due to a mixture of spasticity and rigidity. The only clinical symptoms overlapping with Salla disease are in fact early psychomotor retardation and speech problems, which by itself is not very disease specific. MRI findings further support the milder clinical phenotype with weak features of hypomyelination and thinning of corpus callosum in contrast to Salla patients where cerebral and cerebellar atrophy, hypomyelination and corpus callosum hypoplasia are typical findings. Thus, the patient described in this paper shows an even milder clinical phenotype of SASD than Salla disease, which makes it difficult to pinpoint the correct diagnosis. Our findings suggest that analysis of free sialic acid needs to be considered in patients with encephalopathy and mild hypomyelination and thinning of corpus callosum.

The elevation of free urinary sialic acid has been considered the biochemical hallmark of SASD and has been observed as early as 3 days of age [27]. Thus, the levels of total urinary sialic acid (free and bound) is elevated in these patients and colorimetric measurement of this fraction is commonly used as the first screening biomarker for SASD and other lysosomal diseases storing sialylated oligosaccharides. In our experience, the vast majority of SASD patients show increased total sialic acid in urine. However, the patient described here shows borderline total sialic acid concentrations at two separate sampling occasions. As a complement to the quantitative assay, oligosaccharide screening in urine samples is routinely performed by thin-layer chromatography which in this case was suggestive of increased levels of free sialic acid. This was confirmed by quantitative measurements of free sialic acid in urine and fibroblasts. The increase of free sialic acid was somewhat lower than previously described Salla patients [27]. These results further highlight the importance of using qualitative analysis of urine oligosaccharides combined with quantitative analysis of total sialic acid. Another possibility to overcome these problems is to use mass spectrometry and simultaneously measure total and free sialic acid [28, 29] which might be the golden standard in the near future. It should also be mentioned that SASD has been reported in two siblings without sialuria, both homozygous for the Lys136Glu mutation in *SLC17A5* [30]. Increased free sialic acid was present in CSF, detected by H-NMR spectroscopy, and this finding together with hypomyelination was suggestive of SASD.

In view of the unusual phenotype in our patient, further investigation of *SLC17A5* was driven by the elevation of free sialic acid in urine and fibroblasts. In the first attempt to find pathogenic variants, Sanger sequencing of the coding regions of *SLC17A5* was performed with negative results of all exons except for exon 9 which could not be amplified. Thus, an untargeted diagnostic approach was used to find out the cause of the elevated SASD biomarker by including the family in the TIDEX gene discovery study. WES analysis identified 20 candidate genes affected by rare variants predicted to affect protein function, however none of these variants was deemed a good explanation for the observed phenotype in the patient. Interestingly, looking more closely into the *SLC17A5* gene, the WES analysis revealed the presence of a homozygous insertion of an unknown size or origin in intron 9. RT-PCR and Sanger re-sequencing further confirmed the presence of a 6040 bp insertion (RefSeq KF425758.1), which was located in intron 9 of *SLC17A5* (24 bp downstreamof exon 9) and showed this insertion to be a LINE-1 retrotransposon [17]. The presence of a large insertion in intron 9 might explain the problems of amplifying exon 9 by the initial Sanger sequencing. This large transposon insertion has previously been described in *SLC25A13* causing citrin deficiency [15]. Furthermore, retrotranspositional insertion of L1 elements resulting in genomic deletions has been shown to cause pyruvate dehydrogenase complex deficiency [31].

Whole exome sequencing profiles only a small portion of the human genome (~1%) by capturing the protein-coding sequences, and one of the disadvantages of this approach is that a ‘pathogenic’ variant may be located outside of the captured region, e.g. missed exonic regions, deep intronic variants, regulatory elements or in other non-coding regions of the genome. Moreover, WES data is not optimal for detection of larger variants, such as copy number variants and structural variants. Using whole genome sequencing (WGS) instead of WES can overcome these problems since WGS refers to analysis of the entire human genome [32] (and references therein). In our patient, the transposon insertion occurred relatively close (within 24 bp) to the exon-intron boundary and we were able to detect it using WES, highlighting the importance of thorough analysis and interpretation of NGS data [11] as well as further molecular analyses to validate the findings discovered using NGS data in patients with suspected genetic disorders and persistent unique biochemical phenotypes.

The possible consequences of this homozygous insertion in the index were investigated through *in silico* analyses. These analyses predicted that the insertion of this sequence 24 bp into intron 9 created two splice sites, occurring 82 bp and 679 bp into the insertion sequence, in addition to the normal splice site between exon 9 and intron 9. cDNA analyses confirmed the presence of three *SLC17A5* transcripts in the index providing support for the presence of all three splice sites in this region. *In silico* translation of the two transcripts created by the alternate splice sites predicts a premature stop codon 4 bp into intron 9 and truncation at amino acid 421. The sialin protein consists of 12 transmembrane regions and the variant found in our patient should result in the absence of two transmembrane domains at the carboxyl end of the protein.

The 32 mutations previously defined in *SLC17A5* [33] show a wide spectrum (missense and nonsense mutations, splice-site mutations, insertions and deletions) and no direct mutational hot-spots have been suggested. The phenotypic variation observed in SASD seems to correlate, to some extent, with the presence of the Arg39Cys mutation [7, 8], where this variant seems to cause a more preserved sialin function compared to other mutations found in compound heterozygous patients. Thus, other *SLC17A5* gene mutations that have more damaging effects on the sialin protein function are suggested to cause ISSD. However, other reports challenge this hypothesis. For example, homozygosity of the Lys136Glu variant has been found to cause both severe Salla disease [23] and a mild SASD phenotype [30]. Landau *et al* furthermore report a phenotypic variability of SASD in affected patients of a single inbred kindred with the same homozygous missense mutation [34]. These findings suggest that polymorphisms in *SLC17A5* or other genes involved in the metabolism of free sialic acid may account for the phenotypic variability. Mutations resulting in the deletion of exons 10 and 11 have previously been reported to have severe consequences [35], which contrasts the milder phenotype in our patient. The case discussed in here presents an interesting explanation for the milder phenotype that is observed, even though it may be presumed that exon 10 and 11 cannot be translated. We therefore propose the following explanation: In our patient, in addition to the two new splice sites resulting in aberrant transcription, we did identify normal transcript which is the result of the retention of the normal splice site. The presence of low levels of wild type protein may provide some capacity to transport sialic acid, although not enough to keep the patient healthy.

## Conclusions

We report for the first time a patient with SASD caused by a large intronic transposon insertion in the *SLC17A5* gene. Through careful analysis of whole exome, cDNA and gDNA sequencing results we were able to characterize the effects of the complex variation identified in this patient. We provide an explanation for the mild clinical presentation that was observed in this patient, which is somewhat different to the phenotypes observed in classical Mb Salla patients. In fact, SASD could be underdiagnosed because of their sometimes non-specific clinical findings. Lysosomal SASD should be considered and included in the differential diagnosis of developmental delay with postnatal onset and signs of white matter disease with hypomyelination.

## Additional file

Additional file 1: Predicted sequences resulting from the LINE-1 retrotransposon insertion. (PDF 162 kb)

## Abbreviations

ISSD: Infantile sialic acid storage disease
LINE-1 (L1): Long interspersed element-1
NGS: Next generation sequencing
SASD: Sialic acid storage disease
WES: Whole exome sequencing
WGS: Whole genome sequencing
