# Supplementary material for: Identification of a large intronic transposal insertion in SLC17A5 causing sialic acid storage disease
Source: Orphanet J Rare Dis. 2017 Feb 10;12:28. doi: 10.1186/s13023-017-0584-6 (PMC5303239; doi:10.1186/s13023-017-0584-6)
Supplement: Additional file 1: — Predicted sequences resulting from the LINE-1 retrotransposon insertion. (PDF 162 kb) [file 13023_2017_584_MOESM1_ESM.pdf]

SUPPLEMENTARY DATA

Predicted cDNA sequences resulting from the LINE-1 L1-HS retrotransposon insertion
The insertion of a 6 kb LINE-1 L1-HS retrotransposon in intron 9 of *SLC17A5* is predicted to create a further two splice sites within this insertion, in addition to the splice site at the exon-intron boundary. The resulting cDNA sequences for these three predicted transcripts are provided below. Untranslated sequences are indicated in grey, translated sequences are indicated in black and stop codons are indicated in red. The location of the primers used in the cDNA analyses is underlined.

SLC17A5 cDNA sequence – Splice site 1 (at the exon-intron boundary)

--**5’ UTR**--
GGCTCACTTTGCGCCAATCCTACGAGAACTCCCAGAACTCCGCTTCCCTAGTCCAACCCAAGCCAGAGTTGCCCACACCTAAGATG
GCGGCGGGGGCGGAGTCGGCGCGGCCGCCTCTGGGCGGACCGCGGGGACTAGACGTGGCCGCGGGGCGGTGTCATCGCCCC
CGCCCCGCCCGGTCCAGCCAGCTCGGCCCGGGGGCTTCGGGCTGTCGGGCCGGCGCTCCCTTCTCTGCCAGGTGGCGAGTACACC
TGCTCACGTAGGCGTC

--**Exon 1**--
ATG AGG TCT CCG GTT CGA GAC CTG GCC CGG AAC GAT GGC GAG GAG AGC ACG GAC CGC ACG CCT CTT CTA CCG
GGC GCC CCA CGG GCC GAA GCC G

--**Exon 2**--
CT CCA GTG TGC TGC TCT GCT CGT TAC AAC TTA GCA ATT TTG GCC TTT TTT GGT TTC TTC ATT GTG TAT GCA TTA CGT
GTG AAT CTG AGT GTT GCG TTA GTG GAT ATG GTA GAT TCA AAT ACA ACT TTA GAA GAT AAT AGA ACT TCC AAG GCG
TGT CCA GAG CAT TCT GCT CCC ATA AAA GTT CAT CAT AAT CAA ACG

--**Exon 3**--
GGT AAG AAG TAC CAA TGG GAT GCA GAA ACT CAA GGA TGG ATT CTC GGT TCC TTT TTT TAT GGC TAC ATC ATC ACA
CAG ATT CCT GGA GGA TAT GTT GCC AGC AAA ATA GGG GGG AAA ATG CTG CTA GGA TTT GGG ATC CTT GGC ACT GCT
GTC CTC ACC CTG TTC ACT CCC ATT GCT GCA GAT TTA GGA GTT GGA CCA CTC ATT GTA CTC AGA GCA CTA GAA GGA
CTA GGA GAG

--**Exon 4**--
GGT GTT ACA TTT CCA GCC ATG CAT GCC ATG TGG TCT TCT TGG GCT CCC CCT CTT GAA AGA AGC AAA CTT CTT AGC
ATT TCA TAT GCA G

--**Exon 5**--
GA GCA CAG CTT GGG ACA GTA ATT TCT CTT CCT CTT TCT GGA ATA ATT TGC TAC TAT ATG AAT TGG ACT TAT GTC TTC
TAC TTT TTT G

--**Exon 6**--
GT ACT ATT GGA ATA TTT TGG TTT CTT TTG TGG ATC TGG TTA GTT AGT GAC ACA CCA CAA AAA CAC AAG AGA ATT TCC
CAT TAT GAA AAG GAA TAC ATT CTT TCA TCA TTA AGA AAT CAG

--**Exon 7**--
CTT TCT TCA CAG AAG TCA GTG CCG TGG GTA CCC ATT TTA AAA TCC CTG CCA CTT TGG GCT ATC GTA GTT GCA CAC
TTT TCT TAC AAC TGG ACT TTT TAT ACT TTA TTG ACA TTA TTG CCT ACT TAT ATG AAG GAG ATC CTA AGG TTC AAT GTT
CAA GAG

--**Exon 8**--
AAT GGG TTT TTA TCT TCA TTG CCT TAT TTA GGC TCT TGG TTA TGT ATG ATC CTG TCT GGT CAA GCT GCT GAC AAT TTA
AGG GCA AAA TGG AAT TTT TCA ACT TTA TGT GTT CGC AGA ATT TTT AGC CTT ATA G

--**Exon 9**--
GA ATG ATT GGA CCT GCA GTA TTC CTG GTA GCT GCT GGC TTC ATT GGC TGT GAT TAT TCT TTG GCC GTT GCT TTC CTA
ACT ATA TCA ACA ACA CTG GGA GGC TTT TGC TCT TCT GGA TTT AGC ATC AAC CAT CTG GAT ATT GCT CCT TC

--**Exon 10**--
G TAT GCT GGT ATC CTC CTG GGC ATC ACA AAT ACA TTT GCC ACT ATT CCA GGA ATG GTT GGG CCC GTC ATT GCT AAA AGT CTG ACC CCT GAT

--**Exon 11**--
AAC ACT GTT GGA GAA TGG CAA ACC GTG TTC TAT ATT GCT GCT GCT ATT AAT GTT TTT GGT GCC ATT TTC TTT ACA CTA TTC GCC AAA GGT GAA GTA CAA AAC TGG GCT CTC AAT GAT CAC CAT GGA CAC AGA CAC **TGA**

--**3' UTR**--
AGGAACCAATAAATAATCCTGCCTCTATTAATGTATTTTTATTTATCATGTAACCTCAAAGTGCCTTCTGTATTGTGTAAGCATTCTA
TGTCTTTTTTTAATTGTACTTGTATTAGATTTTTAAGGCCTATAATCATGAAATATCACTAGTTGCCAGAATAATAAAATGAACTGTG
TTTAATTATGAATAATATGTAAGCTAGGACTTCTACTTTAGGTTCACATACCTGCCTGCTAGTCGGGCAACATGAAGTAGGACAGT
TCTGTTGATTTTTTAGGGCCATACTAAAGGGAATGAGCTGAAACAGACCTCCTGATACCTTTGCTTAATTAAACTAGATGATAATTC
TCAGGTACTGATAAACACCTGTTGTTGTTCACTTTCCTCATAAAAATTGTCAGCTCTCTCTGACACTTAGACCTCAAACTTTAGCATC
TCTGTGGAGCTGCCATCCACTGTATAATTTCGCCTGGCAACTGGACTGAGGGGAGTGTGCCCAGGCAGCTGCCAAGCACTCCCTC
CCTGGCTTCAGGGTCAGAGTGCCCAGCGTTTATCAGAGGCAGCATCCAAGCCCAGAGCCAGTGTCGACTCTTCGGCTGGTGCCTT
TCCTCTGAGGGGCTATCAATGTGTAGATAAAGCCCTGAGTAGGCAAGAGCAGTGAGATCCACTGCTATGGTCTTGATACATCCTC
AAACTTTCCCTTCCCAGCACAGAGGAATATTGGCTGGCATGCAACCTGCAAAAGAAAAATGCGAAGCGGCCGGGCACGGTGGCT
CATGCCTGTAATCCCAGCACTTTGGGGGGCTGAGGTGGGCGAATCATGAGATCAGGAGTTCGAGACCAGCCTGGCCAGCATGGT
GAAACCCCATCTCTACTAAAAATACAAAAAATTAGCTGGGCGTGGTGACGGGCGCCTGTAATCCCAGATACTCAGGAGGCTGAGG
TAGGAGAATCACTTGAACCTGGGAGGTGGAAGTTGCAGTGAACCAAGATCACGCCACTGCACTCCAGCCTGGGCGATGGAGCGA
GACTCCAACTCAAAAAAAAAAAAAAGAATAAAGAAAGAAAAGTGCGATGCCCAGTCAATCACAAATAAGATCATCCTGGTTTAAAT
CTACTCTCACATGGATCACAGTATAAATTTCTATGTGCTGTGTTTTGTTCGTTTGTATTTTGTAGAGATGGGGTCTCGTTTTGTCGCC
CAGGCTGGTTTTGAACTCCTGGCTTCAAGCGATCCTCCTGTCTCGGCCTCACAAAGTGTTGAGACTACAGGCATGAGCCACTGTGC
CCAGCCTGTTCTATGTTTTTAAGCTACACGAGAATTTTTTTTTTAATTAATTCTCACTGTTTGTTCAGTCTGTCTTCATCTAAGTTTGT
GTTGCAGTTTAAAGTTAAAGTGACTTTTAAAGGCCACATCACCTGAGACTAGGGTAATCATCTTTACTTCTGGTTCCTGAAATCATA
TTTTTCCAGTGGACCATCCTCCAGTGGCTGTGGTTGTTGAGCATGCTTTCAGAACACCTATGTGGCTTAAAACTTAGTTTATGTTTT
GTGTTCAACACTACGTGTAATATTTTAAAACTGTTTAATGTGATGTGAATACATTTATGTACATTTATTTTTAAATTTGTAAATAGCT
TTAAATTGCTATGGCAATGTTTCTTTTATAAATCATCAAAATAAACCTTTGTGAATTGA

The predicted amplicon size for the exon 9-11 cDNA PCR for this transcript is 517 bp

SLC17A5 cDNA sequence – Splice site 2 (82 bp into the insertion sequence)

--**5' UTR**--
GGCTCACTTTGCGCCAATCCTACGAGAACTCCCAGAACTCCGCTTCCCTAGTCCAACCCAAGCCAGAGTTGCCCACACCTAAGATG
GCGGCGGGGGCGGAGTCGGCGCGGCCGCCTCTGGGCGGGACCGCGGGGACTAGACGTGGCCGCGGGGCGGTGTCATCGCCCC
CGCCCCGCCCGGTCCAGCCAGCTCGGCCCGGGGGCTTCGGGCTGTCGGGCCGGCGCTCCCTTCTCTGCCAGGTGGCGAGTACACC
TGCTCACGTAGGCGTC

--**Exon 1**--
ATG AGG TCT CCG GTT CGA GAC CTG GCC CGG AAC GAT GGC GAG GAG AGC ACG GAC CGC ACG CCT CTT CTA CCG GGC GCC CCA CGG GCC GAA GCC G

--**Exon 2**--
CT CCA GTG TGC TGC TCT GCT CGT TAC AAC TTA GCA ATT TTG GCC TTT TTT GGT TTC TTC ATT GTG TAT GCA TTA CGT GTG AAT CTG AGT GTT GCG TTA GTG GAT ATG GTA GAT TCA AAT ACA ACT TTA GAA GAT AAT AGA ACT TCC AAG GCG TGT CCA GAG CAT TCT GCT CCC ATA AAA GTT CAT CAT AAT CAA ACG

--**Exon 3**--
GGT AAG AAG TAC CAA TGG GAT GCA GAA ACT CAA GGA TGG ATT CTC GGT TCC TTT TTT TAT GGC TAC ATC ATC ACA CAG ATT CCT GGA GGA TAT GTT GCC AGC AAA ATA GGG GGG AAA ATG CTG CTA GGA TTT GGG ATC CTT GGC ACT GCT GTC CTC ACC CTG TTC ACT CCC ATT GCT GCA GAT TTA GGA GTT GGA CCA CTC ATT GTA CTC AGA GCA CTA GAA GGA CTA GGA GAG

--**Exon 4**--
GGT GTT ACA TTT CCA GCC ATG CAT GCC ATG TGG TCT TCT TGG GCT CCC CCT CTT GAA AGA AGC AAA CTT CTT AGC ATT TCA TAT GCA G

---Exon 5---

GA GCA CAG CTT GGG ACA GTA ATT TCT CTT CCT CTT TCT GGA ATA ATT TGC TAC TAT ATG AAT TGG ACT TAT GTC TTC TAC TTT TTT G

---Exon 6---

GT ACT ATT GGA ATA TTT TGG TTT CTT TTG TGG ATC TGG TTA GTT AGT GAC ACA CCA CAA AAA CAC AAG AGA ATT TCC CAT TAT GAA AAG GAA TAC ATT CTT TCA TCA TTA AGA AAT CAG

---Exon 7---

CTT TCT TCA CAG AAG TCA GTG CCG TGG GTA CCC ATT TTA AAA TCC CTG CCA CTT TGG GCT ATC GTA GTT GCA CAC TTT TCT TAC AAC TGG ACT TTT TAT ACT TTA TTG ACA TTA TTG CCT ACT TAT ATG AAG GAG ATC CTA AGG TTC AAT GTT CAA GAG

---Exon 8---

AAT GGG TTT TTA TCT TCA TTG CCT TAT TTA GGC TCT TGG TTA TGT ATG ATC CTG TCT GGT CAA GCT GCT GAC AAT TTA AGG GCA AAA TGG AAT TTT TCA ACT TTA TGT GTT CGC AGA ATT TTT AGC CTT ATA G

---Exon 9---

GA ATG ATT GGA CCT GCA GTA TTC CTG GTA GCT GCT GGC TTC ATT GGC TGT GAT TAT TCT TTG GCC GTT GCT TTC CTA ACT ATA TCA ACA ACA CTG GGA GGC TTT TGC TCT TCT GGA TTT AGC ATC AAC CAT CTG GAT ATT GCT CCT TC

--Intron 9--

G **TGA** gtactaatataaagatttgc

--Insertion--

atggccgaataggaacagctccggtctacagctcccagcgtgagcgacgcagaagacggtgatttctgcatttccatctgag

---Exon 10---

GTATGCTGGTATCCTCCTGGGCATCACAAATACATTTGCCACTATTCCAGGAATGGTTGGGCCCGTCATTGCTAAAAGTCTGACCC CTGAT

---Exon 11---

AACACTGTTGGAGAATGGCAAACCGTGTTCTATATTGCTGCTGCTATTAATGTTTTTGGTGCCATTTTCTTTACACTATTCGCCAAA GGTGAAGTACAAAACTGGGCTCTCAATGATCACCATGGACACAGACACTGA

---3' UTR---

AGGAACCAATAAATAATCCTGCCTCTATTAATGTATTTTTATTTATCATGTAACCTCAAAGTGCCTTCTGTATTGTGTAAGCATTCTA
TGTCTTTTTTTAATTGTACTTGTATTAGATTTTTAAGGCCTATAATCATGAAATATCACTAGTTGCCAGAATAATAAAATGAACTGTG
TTTAATTATGAATAATATGTAAGCTAGGACTTCTACTTTAGGTTCACATACCTGCCTGCTAGTCGGGCAACATGAAGTAGGACAGT
TCTGTTGATTTTTTAGGGCCATACTAAAGGGAATGAGCTGAAACAGACCTCCTGATACCTTTGCTTAATTAAACTAGATGATAATTC
TCAGGTACTGATAAACACCTGTTGTTGTTCACTTTCCTCATAAAAATTGTCAGCTCTCTCTGACACTTAGACCTCAAACTTTAGCATC
TCTGTGGAGCTGCCATCCACTGTATAATTTCGCCTGGCAACTGGACTGAGGGGAGTGTGCCCAGGCAGCTGCCAAGCACTCCCTC
CCTGGCTTCAGGGTCAGAGTGCCCAGCGTTTATCAGAGGCAGCATCCAAGCCCAGAGCCAGTGTCGACTCTTCGGCTGGTGCCTT
TCCTCTGAGGGGCTATCAATGTGTAGATAAAGCCCTGAGTAGGCAAGAGCAGTGAGATCCACTGCTATGGTCTTGATACATCCTC
AAACTTTCCCTTCCCAGCACAGAGGAATATTGGCTGGCATGCAACCTGCAAAAGAAAAATGCGAAGCGGCCGGGCACGGTGGCT
CATGCCTGTAATCCCAGCACTTTGGGGGGCTGAGGTGGGCGAATCATGAGATCAGGAGTTCGAGACCAGCCTGGCCAGCATGGT
GAAACCCCATCTCTACTAAAAATACAAAAAATTAGCTGGGCGTGGTGACGGGCGCCTGTAATCCCAGATACTCAGGAGGCTGAGG
TAGGAGAATCACTTGAACCTGGGAGGTGGAAGTTGCAGTGAACCAAGATCACGCCACTGCACTCCAGCCTGGGCGATGGAGCGA
GACTCCAACTCAAAAAAAAAAAAAAGAATAAAGAAAGAAAAGTGCGATGCCCAGTCAATCACAAATAAGATCATCCTGGTTTAAAT
CTACTCTCACATGGATCACAGTATAAATTTCTATGTGCTGTGTTTTGTTCGTTTGTATTTTGTAGAGATGGGGTCTCGTTTTGTCGCC
CAGGCTGGTTTTGAACTCCTGGCTTCAAGCGATCCTCCTGTCTCGGCCTCACAAAGTGTTGAGACTACAGGCATGAGCCACTGTGC
CCAGCCTGTTCTATGTTTTTAAGCTACACGAGAATTTTTTTTTTAATTAATTCTCACTGTTTGTTCAGTCTGTCTTCATCTAAGTTTGT
GTTGCAGTTTAAAGTTAAAGTGACTTTTAAAGGCCACATCACCTGAGACTAGGGTAATCATCTTTACTTCTGGTTCCTGAAATCATA
TTTTTCCAGTGGACCATCCTCCAGTGGCTGTGGTTGTTGAGCATGCTTTCAGAACACCTATGTGGCTTAAAACTTAGTTTATGTTTT
GTGTTCAACACTACGTGTAATATTTTAAAACTGTTTAATGTGATGTGAATACATTTATGTACATTTATTTTTAAATTTGTAAATAGCT
TTAAATTGCTATGGCAATGTTTCTTTTATAAATCATCAAAATAAACCTTTGTGAATTGA

The predicted amplicon size for the exon 9-11 cDNA PCR for this transcript is 623 bp

SLC17A5 cDNA sequence – Splice site 3 (679 bp into the insertion sequence)

--**5' UTR**--

GGCTCACTTTGCGCCAATCCTACGAGAACTCCCAGAACTCCGCTTCCCTAGTCCAACCCAAGCCAGAGTTGCCCACACCTAAGATG
GCGGCGGGGGCGGAGTCGGCGCGGCCGCCTCTGGGCGGGACCGCGGGGACTAGACGTGGCCGCGGGGCGGTGTCATCGCCCC
CGCCCCGCCCGGTCCAGCCAGCTCGGCCCGGGGGCTTCGGGCTGTCGGGCCGGCGCTCCCTTCTCTGCCAGGTGGCGAGTACACC
TGCTCACGTAGGCGTC

--**Exon 1**--

ATG AGG TCT CCG GTT CGA GAC CTG GCC CGG AAC GAT GGC GAG GAG AGC ACG GAC CGC ACG CCT CTT CTA CCG
GGC GCC CCA CGG GCC GAA GCC G

--**Exon 2**--

CT CCA GTG TGC TGC TCT GCT CGT TAC AAC TTA GCA ATT TTG GCC TTT TTT GGT TTC TTC ATT GTG TAT GCA TTA CGT
GTG AAT CTG AGT GTT GCG TTA GTG GAT ATG GTA GAT TCA AAT ACA ACT TTA GAA GAT AAT AGA ACT TCC AAG GCG
TGT CCA GAG CAT TCT GCT CCC ATA AAA GTT CAT CAT AAT CAA ACG

--**Exon 3**--

GGT AAG AAG TAC CAA TGG GAT GCA GAA ACT CAA GGA TGG ATT CTC GGT TCC TTT TTT TAT GGC TAC ATC ATC ACA
CAG ATT CCT GGA GGA TAT GTT GCC AGC AAA ATA GGG GGG AAA ATG CTG CTA GGA TTT GGG ATC CTT GGC ACT GCT
GTC CTC ACC CTG TTC ACT CCC ATT GCT GCA GAT TTA GGA GTT GGA CCA CTC ATT GTA CTC AGA GCA CTA GAA GGA
CTA GGA GAG

--**Exon 4**--

GGT GTT ACA TTT CCA GCC ATG CAT GCC ATG TGG TCT TCT TGG GCT CCC CCT CTT GAA AGA AGC AAA CTT CTT AGC
ATT TCA TAT GCA G

--**Exon 5**--

GA GCA CAG CTT GGG ACA GTA ATT TCT CTT CCT CTT TCT GGA ATA ATT TGC TAC TAT ATG AAT TGG ACT TAT GTC TTC
TAC TTT TTT G

--**Exon 6**--

GT ACT ATT GGA ATA TTT TGG TTT CTT TTG TGG ATC TGG TTA GTT AGT GAC ACA CCA CAA AAA CAC AAG AGA ATT TCC
CAT TAT GAA AAG GAA TAC ATT CTT TCA TCA TTA AGA AAT CAG

--**Exon 7**--

CTT TCT TCA CAG AAG TCA GTG CCG TGG GTA CCC ATT TTA AAA TCC CTG CCA CTT TGG GCT ATC GTA GTT GCA CAC
TTT TCT TAC AAC TGG ACT TTT TAT ACT TTA TTG ACA TTA TTG CCT ACT TAT ATG AAG GAG ATC CTA AGG TTC AAT GTT
CAA GAG

--**Exon 8**--

AAT GGG TTT TTA TCT TCA TTG CCT TAT TTA GGC TCT TGG TTA TGT ATG ATC CTG TCT GGT CAA GCT GCT GAC AAT TTA
AGG GCA AAA TGG AAT TTT TCA ACT TTA TGT GTT CGC AGA ATT TTT AGC CTT ATA G

--**Exon 9**--

GA ATG ATT GGA CCT GCA GTA TTC CTG GTA GCT GCT GGC TTC ATT GGC TGT GAT TAT TCT TTG GCC GTT GCT TTC CTA
ACT ATA TCA ACA ACA CTG GGA GGC TTT TGC TCT TCT GGA TTT AGC ATC AAC CAT CTG GAT ATT GCT CCT TC

--**Intron 9**--

G **TGA** gtactaatataaagatttgc

--**Insertion**--

atggccgaataggaacagctccggtctacagctcccagcgtgagcgacgcagaagacggtgatttctgcatttccatctgaggtaccgggttcatctcactagggagtgcc
agacagtgggcgcaggccagtgtgtgtgcgcaccgtgcgcgagccgaagcagggcgaggcattgcctcacctgggaagcgcaaggggtcagggagttccctttctgagt
caaagaaaggggtgacggtcgcacctggaaaatcgggtcactcccacccgaatattgcgcttttcagaccggcttaagaaacggcgcaccacgagactatatcccacacc
tggctcggagggtcctacgcccacggaatctcgctgattgctagcacagcagtctgagatcaaactgcaaggcggcaacgaggctgggggaggggcgcccgccattgcc
caggcttgcttaggtaaacaaagcagccgggaagctcgaactgggtggagcccaccacagctcaaggaggcctgcctgcctctgtaggctccacctctgggggcagggc
acagacaaacaaaaagacagcagtaacctctgcagacttaagtgtccctgtctgacagctttgaagagagcagtggttctcccagcacgcagctggagatctgagaacg
ggcagacagactgcctcctcaa

--**Exon 10**--

GTATGCTGGTATCCTCCTGGGCATCACAAATACATTTGCCACTATTCCAGGAATGGTTGGGCCCGTCATTGCTAAAAGTCTGACCC
CTGAT

--Exon 11--

AACACTGTTGGAGAATGGCAAACCGTGTTCTATATTGCTGCTGCTATTAATGTTTTTGGTGCCATTTTCTTTACACTATTCGCCAAA
GGTGAAGTACAAAACTGGGCTCTCAATGATCACCATGGACACAGACACTGA

--3' UTR--

AGGAACCAATAAATAATCCTGCCTCTATTAATGTATTTTTATTTATCATGTAACCTCAAAGTGCCTTCTGTATTGTGTAAGCATTCTA
TGTCTTTTTTTAATTGTACTTGTATTAGATTTTTAAGGCCTATAATCATGAAATATCACTAGTTGCCAGAATAATAAAATGAACTGTG
TTTAATTATGAATAATATGTAAGCTAGGACTTCTACTTTAGGTTCACATACCTGCCTGCTAGTCGGGCAACATGAAGTAGGACAGT
TCTGTTGATTTTTTAGGGCCATACTAAAGGGAATGAGCTGAAACAGACCTCCTGATACCTTTGCTTAATTAAACTAGATGATAATTC
TCAGGTACTGATAAACACCTGTTGTTGTTCACTTTCCTCATAAAAATTGTCAGCTCTCTCTGACACTTAGACCTCAAACTTTAGCATC
TCTGTGGAGCTGCCATCCACTGTATAATTTCGCCTGGCAACTGGACTGAGGGGAGTGTGCCCAGGCAGCTGCCAAGCACTCCCTC
CCTGGCTTCAGGGTCAGAGTGCCCAGCGTTTATCAGAGGCAGCATCCAAGCCCAGAGCCAGTGTCGACTCTTCGGCTGGTGCCTT
TCCTCTGAGGGGCTATCAATGTGTAGATAAAGCCCTGAGTAGGCAAGAGCAGTGAGATCCACTGCTATGGTCTTGATACATCCTC
AAACTTTCCCTTCCCAGCACAGAGGAATATTGGCTGGCATGCAACCTGCAAAAGAAAAATGCGAAGCGGCCGGGCACGGTGGCT
CATGCCTGTAATCCCAGCACTTTGGGGGGCTGAGGTGGGCGAATCATGAGATCAGGAGTTCGAGACCAGCCTGGCCAGCATGGT
GAAACCCCATCTCTACTAAAAATACAAAAAATTAGCTGGGCGTGGTGACGGGCGCCTGTAATCCCAGATACTCAGGAGGCTGAGG
TAGGAGAATCACTTGAACCTGGGAGGTGGAAGTTGCAGTGAACCAAGATCACGCCACTGCACTCCAGCCTGGGCGATGGAGCGA
GACTCCAACTCAAAAAAAAAAAAAAGAATAAAGAAAGAAAAGTGCGATGCCCAGTCAATCACAAATAAGATCATCCTGGTTTAAAT
CTACTCTCACATGGATCACAGTATAAATTTCTATGTGCTGTGTTTTGTTCGTTTGTATTTTGTAGAGATGGGGTCTCGTTTTGTCGCC
CAGGCTGGTTTTGAACTCCTGGCTTCAAGCGATCCTCCTGTCTCGGCCTCACAAAGTGTTGAGACTACAGGCATGAGCCACTGTGC
CCAGCCTGTTCTATGTTTTTAAGCTACACGAGAATTTTTTTTTTAATTAATTCTCACTGTTTGTTCAGTCTGTCTTCATCTAAGTTTGT
GTTGCAGTTTAAAGTTAAAGTGACTTTTAAAGGCCACATCACCTGAGACTAGGGTAATCATCTTTACTTCTGGTTCCTGAAATCATA
TTTTTCCAGTGGACCATCCTCCAGTGGCTGTGGTTGTTGAGCATGCTTTCAGAACACCTATGTGGCTTAAAACTTAGTTTATGTTTT
GTGTTCAACACTACGTGTAATATTTTAAAACTGTTTAATGTGATGTGAATACATTTATGTACATTTATTTTTAAATTTGTAAATAGCT
TTAAATTGCTATGGCAATGTTTCTTTTATAAATCATCAAAATAAACCTTTGTGAATTGA

The predicted amplicon size for the exon 9-11 cDNA PCR for this transcript is 1220 bp
